# Prognostic and predictive factors for angiosarcoma patients receiving paclitaxel once weekly plus or minus bevacizumab: an ancillary study derived from a randomized clinical trial

**DOI:** 10.1186/s12885-018-4828-1

**Published:** 2018-10-11

**Authors:** Loïc Lebellec, François Bertucci, Emmanuelle Tresch-Bruneel, Isabelle Ray-Coquard, Axel Le Cesne, Emmanuelle Bompas, Jean-Yves Blay, Antoine Italiano, Olivier Mir, Thomas Ryckewaert, Yves Toiron, Luc Camoin, Anthony Goncalves, Nicolas Penel, Marie-Cécile Le Deley

**Affiliations:** 1 Lille University Hospital and Medical School, 59045 Lille cedex, France; 2 Department of Medical Oncology, Institut Paoli-Calmettes, 232 Boulevard de Sainte-Marguerite, 13009 Marseille, France; 3 Direction of Research and Innovation, Centre Oscar Lambret, 3 rue Combemale, 59020 Lille cedex, France; 4 Department of Medical Oncology, Centre Léon Bérard, 28 Prom. Léa et Napoléon Bullukian, 69008 Lyon, France; 5 Department of Medical Oncology, Gustave Roussy Institute, 114 Rue Edouard Vaillant, 94800 Villejuif, France; 6 Department of Medical Oncology, Centre René Gauducheau, Boulevard Professeur Jacques Monod, 44805 Saint-Herblain, France; 7 Department of Medical Oncology, Institut Bergonié, 229 Cours de l’Argonne, 33000, Bordeaux, France; 8 Department of Molecular Pharmacology, Institute Paoli-Calmettes, 232 Boulevard de Sainte-Marguerite, 13009, Marseille, France; 9 General Oncology Department, Centre Oscar Lambret, Lille, 3 rue Combemale, 59020 Lille cedex, France; 10 Paris-Saclay University, Paris-Sud University, UVSQ, CESP, INSERM, Villejuif, France; 11 Department of Clinical Research and Innovation, Centre Oscar Lambret, 3, rue Combemale, 59020 Lille, France

**Keywords:** Angiosarcoma, Bevacizumab, Biomarkers, Radiation-induced angiosarcoma, Weekly paclitaxel

## Abstract

**Background:**

We report here a correlation analysis conducted along with a phase II trial assessing bevacizumab in combination with weekly paclitaxel.

**Methods:**

Circulating pro/anti-angiogenic factors were assessed on day 1 (D1) and day 8 (D8). The prognostic value for progression-free survival (PFS) was evaluated using a Cox model with biomarkers as continuous variables.

**Results:**

Among the 51 patients enrolled and treated in this trial, biomarker analysis was performed for 42: 18 in Arm A (single-agent) and 24 in Arm B (combination). With a median follow-up of 46 months, PFS was 5.5 versus 5.7 months, respectively (*p* = 0.75). According to univariate analysis, factors associated with a poor PFS were as follows: visceral angiosarcoma, de novo angiosarcoma, and high PlGF and low VEGF-C baseline values. In multivariate analysis, de novo angiosarcoma (HR = 2.5; *p* = 0.024) and baseline VEGF-C value (HR = 0.7; *p* = 0.003) were significant prognostic factors. We observed a significant increase in circulating PlGF (< 0.001) and a decrease in VEGF (< 0.001) during bevacizumab treatment. An increase in FGF was associated with a poor outcome.

**Conclusions:**

De novo angiosarcoma and a low baseline level of VEGF-C were found to be associated with a poor prognosis. Addition of bevacizumab induces major changes in circulating biomarkers (VEGF and PlGF) in a short timeframe without impacting PFS.

**Trial registration:**

Retrospectively registered on EudraCT N° 2009–017020-59 and NCT01303497 (February 24, 2011).

**Electronic supplementary material:**

The online version of this article (10.1186/s12885-018-4828-1) contains supplementary material, which is available to authorized users.

## Background

Angiosarcomas account for approximately 1% of adult soft-tissue sarcomas, the latter of which account for 1–2% of adult malignancies. Angiosarcomas are heterogeneous and involve at least, on the one hand, de novo versus radio-induced sarcoma and, on the other hand, skin or scalp locations versus visceral locations [1]. Regardless, these entities exhibit aggressive behavior, leading to a poor outcome. Localized angiosarcomas are best treated by large en-bloc surgery followed by adjuvant radiotherapy. Locally advanced (not amenable to curative-intent surgery) or metastatic angiosarcomas are treated with palliative chemotherapy aiming to alleviate symptoms and maintain quality of life [1]. Doxorubicin and weekly paclitaxel are both regarded as a preferred option as the first or second line, and these regimens provide a median overall survival of approximately 8 to 12 months [2]. Thus, advanced angiosarcoma treatment remains an unmet medical need.

Assessing the activity of anti-angiogenic agents, such as bevacizumab (a humanized monoclonal antibody against vascular endothelial growth factor (VEGF)), in angiosarcoma is important, and preclinical studies have demonstrated a key role for angiogenesis in angiosarcoma proliferation [3–12]. In general, angiosarcomas overexpress VEGF-A as well as multiple VEGF receptors (VEGFRs), including the major pro-angiogenic VEGF-A receptor VEGFR-2 [3–12]. Some studies have reported recurrent activating mutations in angiogenesis signaling genes, especially VEGF receptors [10], and in other genes encoding proteins associated with regulation of VEGF receptors, such as PTPRB and PLCG1 [13]. In vitro, blockade of the VEGF pathway inhibits tumor growth by decreasing proliferation and increasing apoptosis in tumor cell [10].

We previously assessed the activity and safety of weekly paclitaxel and weekly paclitaxel in combination with bevacizumab for advanced angiosarcoma and found no clinical benefit of adding bevacizumab [14]. The present ancillary analysis aims to identify prognostic and predictive factors in the current trial with a longer follow-up.

## Methods

### Clinical trial

AngioTax-Plus was a multicenter, randomized, stratified and open-label phase II trial. The stratification factors were as follows: de novo versus radiation-induced angiosarcoma and superficial (skin and soft tissue) versus visceral angiosarcoma. The efficacy of the phase II trial were previously reported [14]. Patients aged 18 years and older were considered eligible for the study. All patients had histologically proven metastatic or advanced angiosarcoma, as reviewed by the Pathology Committee of the French Sarcoma Group, and were not amenable to curative-intent surgery. Radiation-induced angiosarcomas were eligible if there was no evidence of recurrence of the prior radiotherapy-treated malignancy. Up to two previous lines of systemic chemotherapy were allowed. Tumors were required to be measurable by computed tomography (CT) or magnetic resonance imaging (MRI), as per RECIST 1.1. In both arms, patients received paclitaxel intravenously at the dose of 90 mg/m^2^ on days 1, 8 and 15 of a 28-day cycle for 6 cycles. In the experimental arm (Arm B), patients received bevacizumab during chemotherapy cycles at the dose of 10 mg/kg every 2 weeks until intolerance or progression. In the absence of disease progression after the 6 cycles of chemotherapy, bevacizumab was administered as maintenance therapy at the dose of 15 mg/kg every 3 weeks until intolerance or progression.

Study investigations were conducted after approval by the local Ethics Committee (Nord-Ouest IV Commit de Protection des Patients) on 9th March, 2010, and after the approval of the French Health Authority (AFSSAPS) on 12th April, 2010. Informed consent was obtained from each patient. The study was registered in the European Clinical Trials Register (EudraCT N° 2009–017020-59) and in the US Clinical Trials Register (NCT01303497) and was conducted in agreement with the Declaration of Helsinki and International Conference on Harmonization of Good Clinical Practice guidelines.

### Biomarker analysis

Blood samples were collected into 5-ml serum-separating tubes (SSTs) on day 1 (D1; baseline) and day 8 (D8). The samples were centrifuged at 3000 rpm for 15 min. The plasma was then transferred to 2 labeled cryotubes and frozen, as soon as possible, at − 80 °C, at each center. The tubes were then transported in containers filled with dry ice to maintain cold chain integrity to the Molecular Pharmacology Laboratory in Paoli-Calmettes Institute, Marseille. Enzyme-linked immunosorbent assay (ELISA) was performed as previously described [15] to measure the following circulating biomarkers: VEGF-C in pg/ml by Quantikine human VEGF Immunoassay (R&D, Minneapolis, USA); sE-Selectin in ng/ml by Human sE-Selectin Immunoassay (R&D, Minneapolis, USA); thrombospondin in ng/ml by Human TSP-1 immunoassay (Neogen, Lexington, USA via interchim); VEGF in ng/ml by Quantikine human VEGF Immunoassay (R&D, Minneapolis, USA); stem-cell factor (SCF) in ng/ml by Quantikine human SCF Immunoassay (R&D, Minneapolis, USA); fibroblast growth factor (FGF) in pg/ml by Quantikine Human FGF basic Immunoassays (R&D, Minneapolis, USA); and placental growth factor (PlGF) in pg/ml by Quantikine Human PlGF Immunoassay (R&D, Minneapolis, USA). Each sample was analyzed in duplicate, and the average value was used for correlations with clinical outcomes.

### Statistical analysis

Biomarker baseline (D1) values were compared between the treatment arms and according to medical history (de novo versus radio-induced sarcoma) using the Wilcoxon and Mann-Whitney tests. In each treatment arm, D8 values were compared with D1 values using the Wilcoxon signed-rank tests for paired data. Differences in biomarker values (D8 – D1) were compared between the treatment arms using linear regression models with adjustment for the D1 values.

The endpoint for the prognostic factor analysis was progression-free survival (PFS), which was defined as the time from randomization to the first progression or death from any cause and analyzed in the entire study population. PFS curves were generated using the Kaplan-Meier method. The impact of covariates on PFS was estimated using Cox models (hazard ratio, HR, and 95% Confidence Interval, 95%CI). The clinical factors investigated were tumor location (superficial versus visceral) and radio-induced sarcoma versus de novo sarcoma. Biomarkers at D1, as well as the variation between D1 and D8, were analyzed as continuous values. The impact of baseline covariates (clinical factors and biomarkers at D1) found to be associated with a *p*-value< 0.20 in univariate analysis were then evaluated in multivariate Cox regression. The significance level was not corrected for multiple testing and was set at 0.05 in this exploratory analysis. For each biomarker, the impact of the (D8-D1) observed variation on the risk of progression was evaluated using Cox models that were adjusted to the baseline value.

The predictive value of each covariate (clinical factor, biomarker at D1, and D8-D1 variation) was investigated using an interaction term between the treatment arm and covariate in a multivariate Cox model including these parameters. The results are illustrated by forest plots in which the biomarkers were categorized as binary variables using the observed median value as the cut-off, as the results were very similar when considering the continuous value or binary variable.

## Results

### Overall description of the study population and PFS

Among the 51 patients enrolled and treated in the clinical trial, samples were collected on D1 for 45, and on D8 for 42. The present report focuses on these 42 patients who were assessable for biomarker analysis at both D1 and D8: 18 patients in arm A (paclitaxel alone) and 24 patients in arm B (paclitaxel and bevacizumab). Details of patient and tumor characteristics, by treatment group, are provided in Table 1. A location map of the tumors is available in Additional file 1: Figure S1. The treatment exposure in the trial and tolerance are provided in Table 2 and the drug-related adverse events (Grade ≥ 3) in Additional file 2: Table S1.

**Table 1:** Baseline patient characteristics

	Arm A (*n* = 18)	Arm B (*n* = 24)	*p*-value
Clinical characteristics			
Female: *n* (%)	13 (72.2)	19 (79.2)	*–*
Age: median (range)	63 (24–80)	65.5 (27–82)	*–*
Radio-induced: *n* (%)	7 (38.9)	12 (50)	*–*
Superficial: *n* (%)^a^	11 (61.1)	16 (66.7)	*–*
Grade 2 or 3: *n* (%)	10 (55.6)	13 (54.2)	*–*
Metastatic: *n* (%)	12 (66.7)	16 (66.7)	*–*
Prior exposure to anthracycline: *n* (%)	8 (44.4)	8 (33.3)	*–*
Performance status (WHO) = 0: *n* (%)^b^	10 (58.8)	13 (54.2)	*–*
Baseline biomarkers (D1)			
Human FGF (pg/mL): mean (sd)	5.6 (2.1)	6.0 (3.5)	0.72
PlGF (pg/mL): mean (sd)	24.7 (17.2)	17.8 (10.2)	0.12
SCF sR/c-kit (ng/mL): mean (sd)	8.9 (2.5)	8.2 (2.6)	0.35
sE-Selectin (ng/mL): mean (sd)	33.7 (18.5)	44.8 (24.9)	0.08
TSP-1 (μg/mL): mean (sd)	54.9 (17.3)	44.9 (21.6)	0.17
VEGF (ng/mL): mean (sd)	0.44 (0.28)	0.55 (0.39)	0.38
VEGF-C (ng/mL): mean (sd)	4.2 (1.5)	4.4 (1.7)	0.70

^a^Superficial angiosarcomas included 19 breast angiosarcomas, 5 skin angiosarcomas, 2 soft-tissue angiosarcomas and 1 scalp angiosarcoma

^b^*WHO* World Health Organization

**Table 2:** Treatment exposure and tolerance

	Arm A (*n = 18*)	Arm B (*n = 24*)	*p***-**value
Cycles of paclitaxel: median (range)	6 (2–6)	6 (1–6)	*–*
Relative dose intensity of paclitaxel: median (range)	0.94 (0.68–1.11)	0.88 (0.55–1.03)	*–*
Number of bevacizumab injections: median (range)	–	9 (1–39)	*–*
Related adverse events (Grade ≥ 3): *n* (%)^a^	2 (11.1)	10 (41.7)	0.03

^a^Graded according to the National Cancer Institute Common Terminology Criteria for Adverse Events, version 4.0

The distribution of baseline values of the seven studied biomarkers did not differ significantly between the treatment arms or between the de novo and radio-induced sarcoma groups (Additional file 3: Figure S2).

**Fig. 1:**
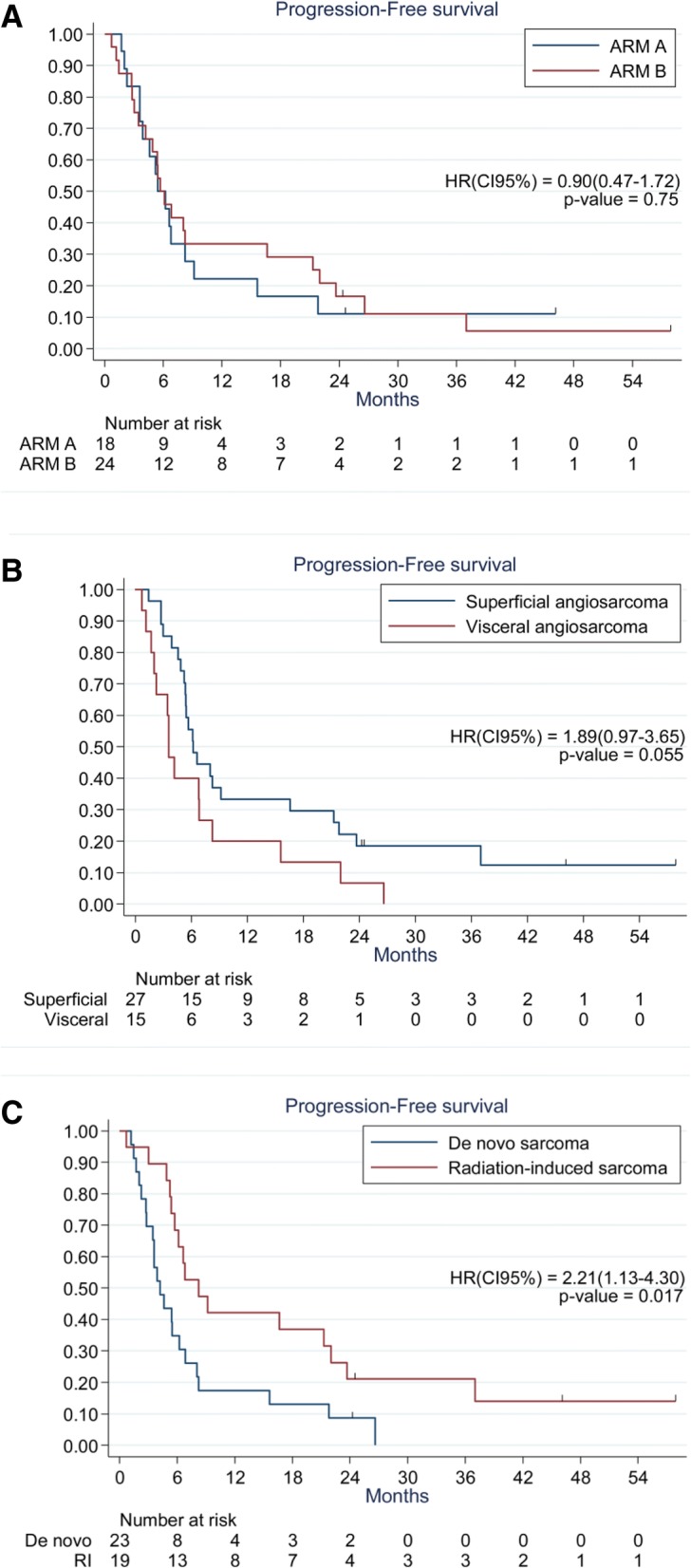
**a** PFS according to treatment arm. **b** PFS according to tumor location. **c** PFS according to radiation-induced or de novo sarcoma

With a median follow-up of 46 months (9.4–57.8), progression was reported in 38 patients (16 in arm A and 22 in arm B), leading to death for 32 patients (13 in arm A and 19 in arm B). The median PFS was 5.5 months (95%CI: 3.6–8.2) in arm A and 5.7 months (95%CI: 3.4–16.6) in arm B. The six-month and 12-month PFS rates were 50% (95%CI: 25.9–70.0) and 22% (95%CI: 6.9–42.9) in arm A and 50% (95%CI: 29.1–67.8) and 33% (95%CI: 15.9–51.9) in arm B, respectively. PFS did not differ significantly between the treatment arms (HR_B/A_ = 0.90; 95%CI, 0.47–1.72; *p = 0.75*; Fig. 1a).

### Prognostic factor analysis at baseline for PFS

In univariate analysis, a non-significant trend for a worse PFS in patients with visceral sarcoma compared with superficial sarcoma was observed (HR = 1.89; 95%CI: 0.97–3.65; *p = 0.055*; Fig. 1b; Table 3). However, the difference was no longer significant in the multivariate model that included medical history and baseline biomarkers (HR = 1.53; 95%CI: 0.73–3.23; *p = 0.26*). PFS was significantly worse in patients with de novo sarcoma than in patients with radiation-induced sarcoma, both in univariate analysis (HR = 2.21; 95%CI: 1.13–4.30; *p = 0.017*; Fig. 1c; Table 3) and in multivariate analysis (HR = 2.49; 95%CI: 1.13–5.50; *p = 0.024*).

**Table 3:** Univariate and multivariate prognostic factor for longer PFS in angiosarcoma patients treated with weekly paclitaxel plus or minus bevacizumab ^a^

Parameters		Univariate analysis			Multivariate analysis^c^		
		HR^b^ (95% CI)		*p*	HR^b^ (95% CI)		*p*
Clinical characteristics							
Location	Superficial	1			1		
	Visceral	1.89	(0.97–3.65)	0.055	1.53	(0.73–3.23)	0.26
Medical history	Radiation-induced	1			1		
	De novo	2.21	(1.13–4.30)	0.017	2.49	(1.13–5.50)	0.024
Baseline biomarkers							
Human FGF (pg/mL)		0.96	(0.86–1.07)	0.48	–		
PlGF (pg/mL)		1.03	(1.00–1.05)	0.037	1.02	(0.99–1.04)	0.17
SCF sR/c-kit (ng/mL)		0.99	(0.87–1.13)	0.86	–		
sE-Selectin (ng/mL)		1.00	(0.98–1.01)	0.85	–		
TSP-1 (μg/mL)		0.99	(0.97–1.01)	0.25	–		
VEGF (ng/mL)		0.70	(0.25–1.96)	0.49	–		
VEGF-C (ng/mL)		0.75	(0.60–0.95)	0.015	0.69	(0.54–0.88)	0.003

^a^Considering the absence of a statistically significant difference, the data from the two arms were pooled

^b^For each biomarker, HR represents the hazard ratio for an increase of one unit

^c^Factors associated with PFS with *p* < 0.20 in univariate analysis were included in the multivariate model: tumor location, medical history, PIGF on D1 and VEGF-C on D1

According to univariate analysis, higher PIGF values and lower VEGF-C values were associated with lower PFS when assessing the seven serum biomarkers listed above (*p = 0.037* and *p = 0.015*, respectively). In multivariate analysis including both biomarkers and clinical factors (Table 3), VEGF-C remained the only biomarker significantly associated with a worse outcome (*p = 0.003*).

### Variation in biomarkers during treatment and impact on PFS

**Fig. 2:**
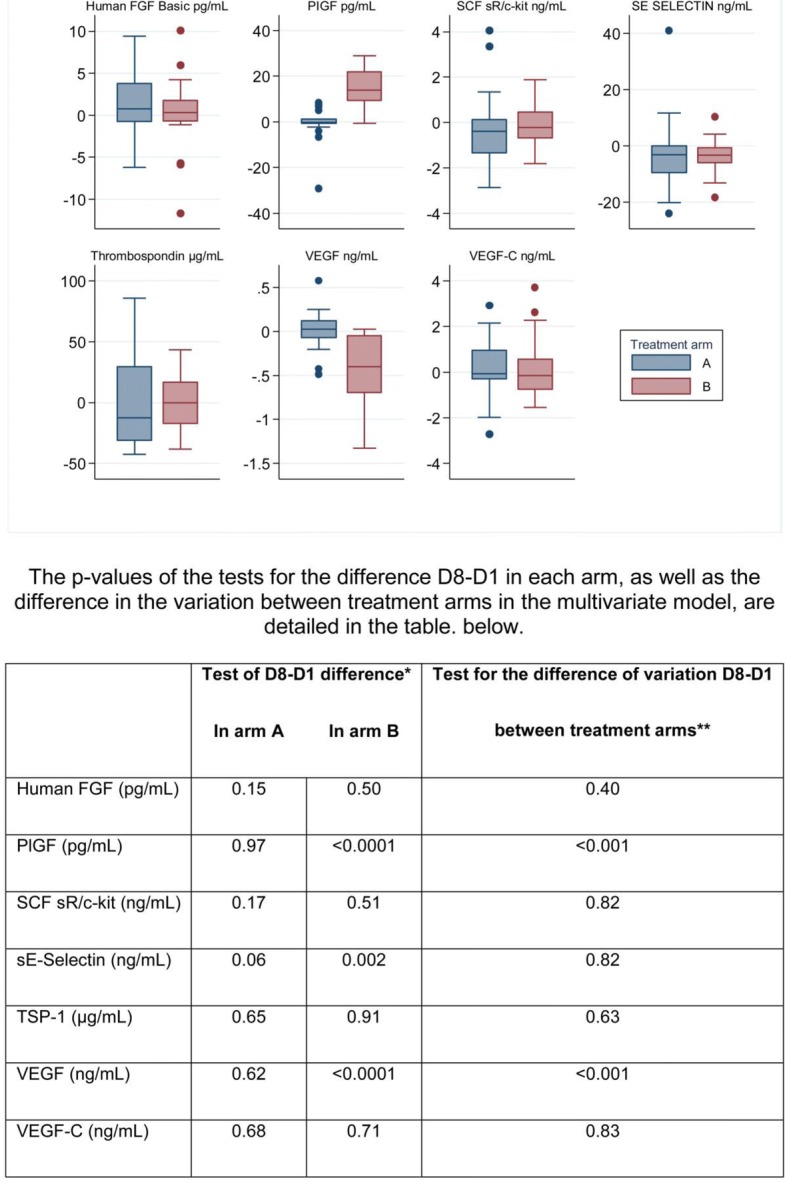
Variation (D8 – D1) in biomarker values in each treatment arm

As detailed in Fig. 2, we observed a significant decrease in the level of VEGF and a significant increase in circulating PlGF between D1 and D8 in arm B (with bevacizumab), whereas the level of these biomarkers remained stable in arm A.

We also observed a slight but significant decrease in sE-Selectin in arm B, though a similar trend was also observed in arm A.

In univariate and multivariate analyses, changes in the level of VEGF and PlGF did not influence PFS, but an increase in FGF was associated with a shorter PFS (Table 4).

**Table 4:** Impact of (D8-D1) variations on PFS in univariate and multivariate analyses^a^

Parameters	Univariate analysis			Multivariate analysis^c^		
	HR^b^ (95% CI)		*p*	HR^b^ (95% CI)		*p*
Variation (D8-D1)						
Human FGF (pg/mL)	1.24	(1.10–1.39)	< 0.001	1.16	(1.03–1.31)	0.016
PlGF (pg/mL)	1.01	(0.98–1.04)	0.51	–		
SCF sR/c-kit (ng/mL)	1.04	(0.74–1.45)	0.83	–		
sE-Selectin (ng/mL)	0.99	(0.96–1.01)	0.46	–		
TSP-1 (μg/mL)	1.00	(0.99–1.01)	0.98	–		
VEGF (ng/mL)	1.30	(0.30–5.65)	0.73	–		
VEGF-C (ng/mL)	1.08	(0.83–1.42)	0.57	–		

^a^Considering the absence of difference statistically significant, the data from the two arms were pooled

^b^For each biomarker, HR represents the hazard ratio for an increase of one unit, as estimated including the baseline value

^c^Factors associated with PFS with *p < 0.20* in univariate analysis were included in a stepwise multivariate model: tumor location, medical history, PIGF on D1, VEGF-C on D1 and variation (D8-D1) of human FGF. Only medical history, VEGF-C on D1 and human FGF variation (D8-D1) were significantly associated with PFS in multivariate analysis. The HR for variation (D8-D1) of human FGF adjusted by medical history and VEGF-C by D1 is presented in the table. The adjusted HRs for medical history and biomarker on D1 were de novo = 2.39 (95% CI: 1.09–5.26), *p = 0.030*, and VEGF-C = 0.73 (0.57–0.94), *p = 0.015,* respectively. Other parameters were associated with PFS with a *p-value > 0.05* and were removed from the model: tumor location (HR = 1.33 (95% CI: 0.61–2.90), *p = 0.47*) and PIGF pg/mL on D1 (HR = 1.00 (95%CI: 0.98–1.03), *p = 0.70*)

### Evaluation of the predictive value of biomarkers on treatment efficacy

As detailed in Table 5 and illustrated in Additional file 4: Figure S3, we did not observe any significant heterogeneity in the treatment effect in terms of PFS across clinical subsets of patients and according to both baseline biomarker values and biomarker variations between D8 and D1. In other words, we did not identify with baseline clinical and biological characteristics any subgroup that benefiting from adding bevacizumab.

**Table 5:** Evaluation of the predictive value of clinical factors and biomarkers for the treatment effect in terms of PFS

Parameters	*p*-value of the interaction term treatment arm x factor^a^
Clinical characteristics	
Location	0.26
Medical history	0.29
Baseline biomarkers	
Human FGF (pg/mL)	0.28
PlGF (pg/mL)	0.57
SCF sR/c-kit (ng/mL)	0.27
sE-Selectin (ng/mL)	0.48
TSP-1 (μg/mL)	0.06
VEGF (ng/mL)	0.82
VEGF-C (ng/mL)	0.77
Variation (D8-D1)	
Human FGF (pg/mL)	0.60
PlGF (pg/mL)	0.67
SCF sR/c-kit (ng/mL)	0.60
sE-Selectin (ng/mL)	0.62
TSP-1 (μg/mL)	0.28
VEGF (ng/mL)	0.45
VEGF-C (ng/mL)	0.93

^a^ *P*-value associated with better outcome when adding bevacizumab

## Discussion

The results of the present study confirmed that adding bevacizumab to weekly paclitaxel did not improve the PFS of advanced angiosarcoma. This study showed that at advanced stages and when treated with weekly paclitaxel, the outcome of radiation-induced angiosarcoma is better than that of de novo angiosarcoma. At baseline, high values of circulating PlGF and low values of VEGF-C were associated with poor outcomes. Treatment with bevacizumab was associated with a significant increase in circulating PlGF and a decrease in circulating VEGF. Finally, an increase in FGF between day 1 and day 8 was associated with a heightened risk of disease progression.

To date, the use of anti-angiogenic antibodies in angiosarcoma patients is somewhat disappointing. The therapeutic role of antiangiogenic antibodies has been assessed in three phase II trials (including the AngioTax-Plus study), and a phase I/II trial has yet to be published. Agulnik et al. conducted a non-randomized phase II trial assessing bevacizumab alone in angiosarcoma patients; the median PFS was 3 months, and the reported best objective response rate was only 8% [16]. In addition, a phase II trial assessing the peptibody trebananib, which inhibits angiopoietin 1 and 2, failed to demonstrate clinical activity and was closed after the first interim analysis (PFS of 1.8 months and best objective response rate of 0%) [17]. The phase I/II trial assessing the combination of the multi-kinase inhibitor pazopanib with TRC 105, an endoglobin antibody, showed convincing signs of activity in angiosarcoma patients, with a median PFS of 5.6 months in nine patients and with two durable complete responses [18]. An ongoing randomized phase III trial is comparing the efficacy of pazopanib alone compared with pazopanib and TRC 105; this trial will formally establish the benefit of adding an endoglobin antibody to the multi-kinase inhibitor **(**NCT02979899). Nonetheless, the present study clearly shows that adding bevacizumab did not improve the outcome of advanced angiosarcoma patients, regardless of clinical or biological characteristics (see Additional file 2: Table S1).

When treated with weekly paclitaxel, the outcome of angiosarcoma patients in advanced stages is better for radiation-induced tumors than it is for de novo angiosarcoma. These findings are consistent with data in the literature. Italiano et al. analyzed the outcome of 117 advanced angiosarcoma patients treated with doxorubicin (*n = 42*) and weekly paclitaxel (*n = 75*) as first-line treatment. These authors reported that the objective response rate was statistically higher in radiation-induced angiosarcoma than in de novo angiosarcoma with doxorubicin (50 versus 15%, *p = 0.03*) or with paclitaxel (74 versus 45%, *p = 0.04*) [19]. In another retrospective study comprising 142 patients treated with different chemotherapy regimens, the outcome of radiation-induced angiosarcoma was slightly better than that of de novo angiosarcoma (median overall survival of 14.3 versus 10.3 months), but significance was not reached (*p = 0.32*) [2]. These data suggest that radiation-induced angiosarcoma is highly sensitive to chemotherapy, though this needs to be confirmed by independent studies.

In the present study, we observed some major changes in biomarker values over a short period of time influenced by the addition of the anti-angiogenic agent. A low level of circulating VEGF-C and a high level of circulating PlGF at baseline were found to be associated with a poor outcome. In a previously published phase II trial assessing the therapeutic role of sorafenib, an oral anti-angiogenic tyrosine kinase inhibitor, we observed baseline PlGF to be associated with a poor outcome in advanced angiosarcoma patients [15]. Moreover, we found a linear correlation between circulating PlGF and time to progression (*p = 0.02*). Of note, Sleijfer et al. described that among sarcoma patients receiving pazopanib, those with increased circulating PlGF experienced a worse outcome with a shorter PFS and overall survival [20]. Nevertheless, the literature data on circulating biomarkers in human angiosarcoma are very spare.

We found that bevacizumab induces a significant decrease in circulating VEGF and an increase in circulating PlGF. To our knowledge, no prior published study has analyzed the impact of bevacizumab on circulating proangiogenic biomarkers in sarcoma patients, especially in angiosarcoma patients. A large body of evidence shows that treatment with bevacizumab induces significant changes in circulating biomarkers in different carcinomas (for example, colo-rectal cancers [21, 22], hepatocarcinoma [23], non-small cell lung cancers [24]), and our observed decrease in circulating VEGF is consistent with bevacizumab’s mechanism of action. Despite the few data on the impact of bevacizumab treatment on circulating PlGF, an increase in PlGF after bevacizumab treatment has been described in the case of non-small cell lung cancer [24] and colo-rectal cancer [25]. In addition, Kopetz et al. described that an increase in circulating FGF may be observed before radiological progression in metastatic colorectal cancer patients treated with bevacizumab [26]. Our findings cannot be further discussed due to the sparse literature data and the notable absence of prior studies assessing the biomarker VEGF network in sarcoma patients treated with bevacizumab.

Regardless, conclusions with respect to the prognostic or predictive values of these changes must be considered with caution owing to the multiple tests performed on this limited sample of patients, the complexity of the circulating pro- and anti-angiogenic factor network, and the fact that negative results can be caused by a lack of power of the tests performed.

## Conclusion

The primary aim of this ancillary study was to identify factors associated with better outcome in patients with advanced angiosarcoma treated with weekly paclitaxel plus or minus bevacizumab. Adding bevacizumab did not improve the outcome of angiosarcoma patients. We found that radiation-induced angiosarcoma is particularly sensitive to weekly paclitaxel compared to de novo angiosarcoma. Ancillary biomarker analysis remains a hypothesis-generating correlation study. Low level of circulating VEGF-C was associated with poor outcome (Table 3). Nevertheless, the main study limitations of the present study is the limited number of cases, collaborative efforts are needed to improve the management of such rare and aggressive subtype of sarcoma.

## Additional files

Additional file 1: **Figure S1.** Location map of the tumors in each treatment arm (A/B). (PPTX 117 kb)

Additional file 2: **Table S1.** All Grade ≥ 3 drug-related adverse events. (DOCX 13 kb)

Additional file 3: **Figure S2.** Baseline biomarker values according to medical history (de novo versus radio-induced angiosarcoma). (PPTX 290 kb)

Additional file 4: **Figure S3.** Forest plot: evaluation of the predictive value of clinical factors and biomarkers for the treatment effect in terms of PFS. Biomarkers were categorized as binary variables using the observed median value as the cut-off to illustrate the results. (PPTX 415 kb)

## Abbreviations

95%CI: 95% Confidence interval
CT: Computed tomography
D1, D8: Day 1, Day 8
ELISA: Enzyme Linked Immuno-Sorbent Assay
FGF: Fibroblast growth factor
HR: Hazard ratio
MRI: Magnetic resonance imaging
PFS: Progression-free survival
PLCG-1: Phospholipase C Gamma 1
PlGF: Placental growth factor
PTPRB: Protein Tyrosine Phosphatase Receptor type B
RECIST: Response evaluation criteria in solid tumors
SCF: Stem-Cell Factor
sd: standard deviation
SST: Serum-Separating Tubes
TSP-1: Thrombospondin-1
VEGF (A/C): Vascular Endothelial Growth Factor (type A/C)
VEGFR: Vascular Endothelial Growth Factor Receptor
WHO: World Health Organization
