# Supplementary figures and images for: Prognostic and predictive factors for angiosarcoma patients receiving paclitaxel once weekly plus or minus bevacizumab: an ancillary study derived from a randomized clinical trial
Source: BMC Cancer. 2018 Oct 11;18:963. doi: 10.1186/s12885-018-4828-1 (PMC6180490; doi:10.1186/s12885-018-4828-1)

## Slide 1
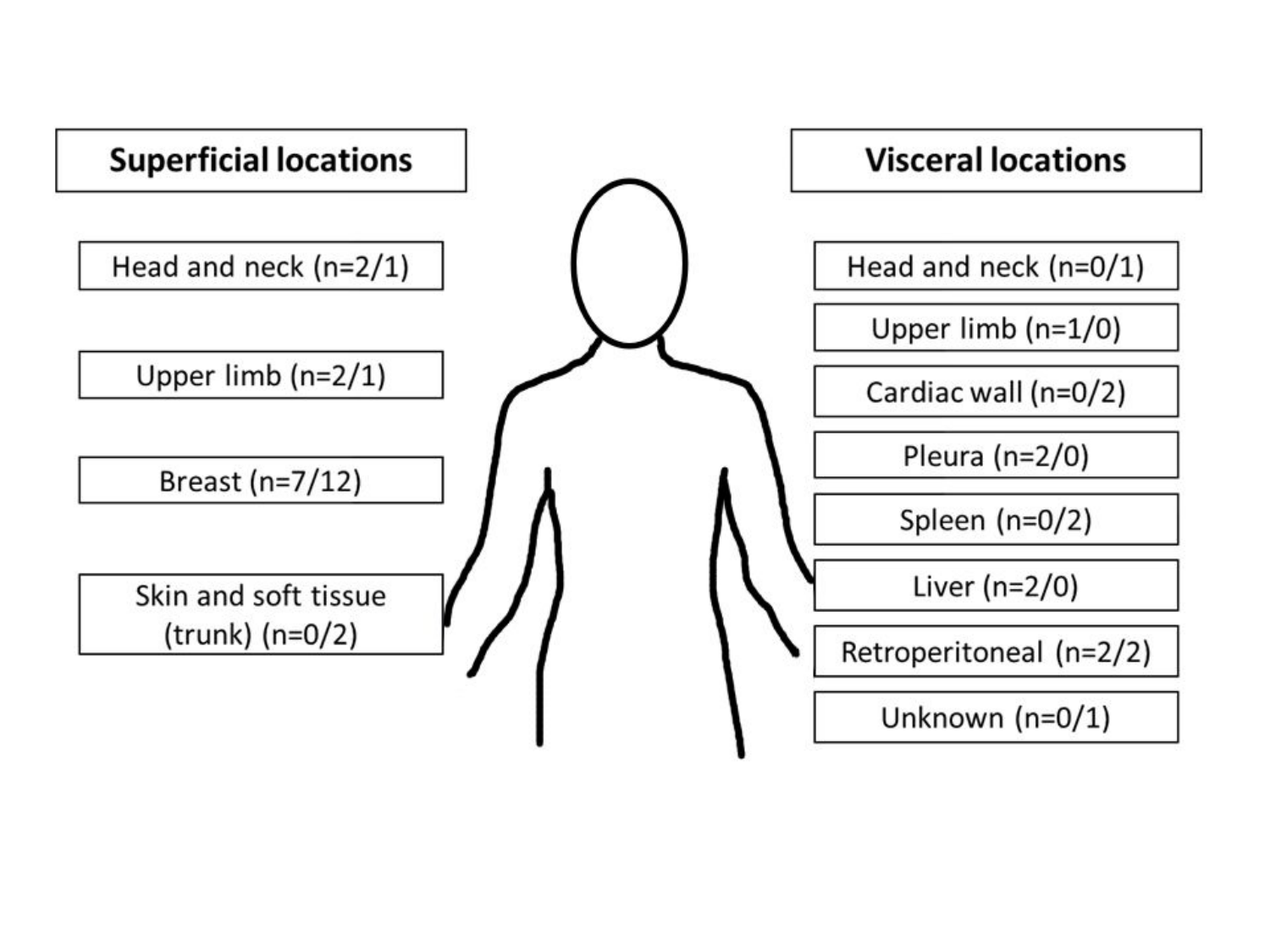

Supplement: Supplementary file 1 — Figure S1. Location map of the tumors in each treatment arm (A/B). (PPTX 117 kb) [file 12885_2018_4828_MOESM1_ESM.pptx]

## Slide 1
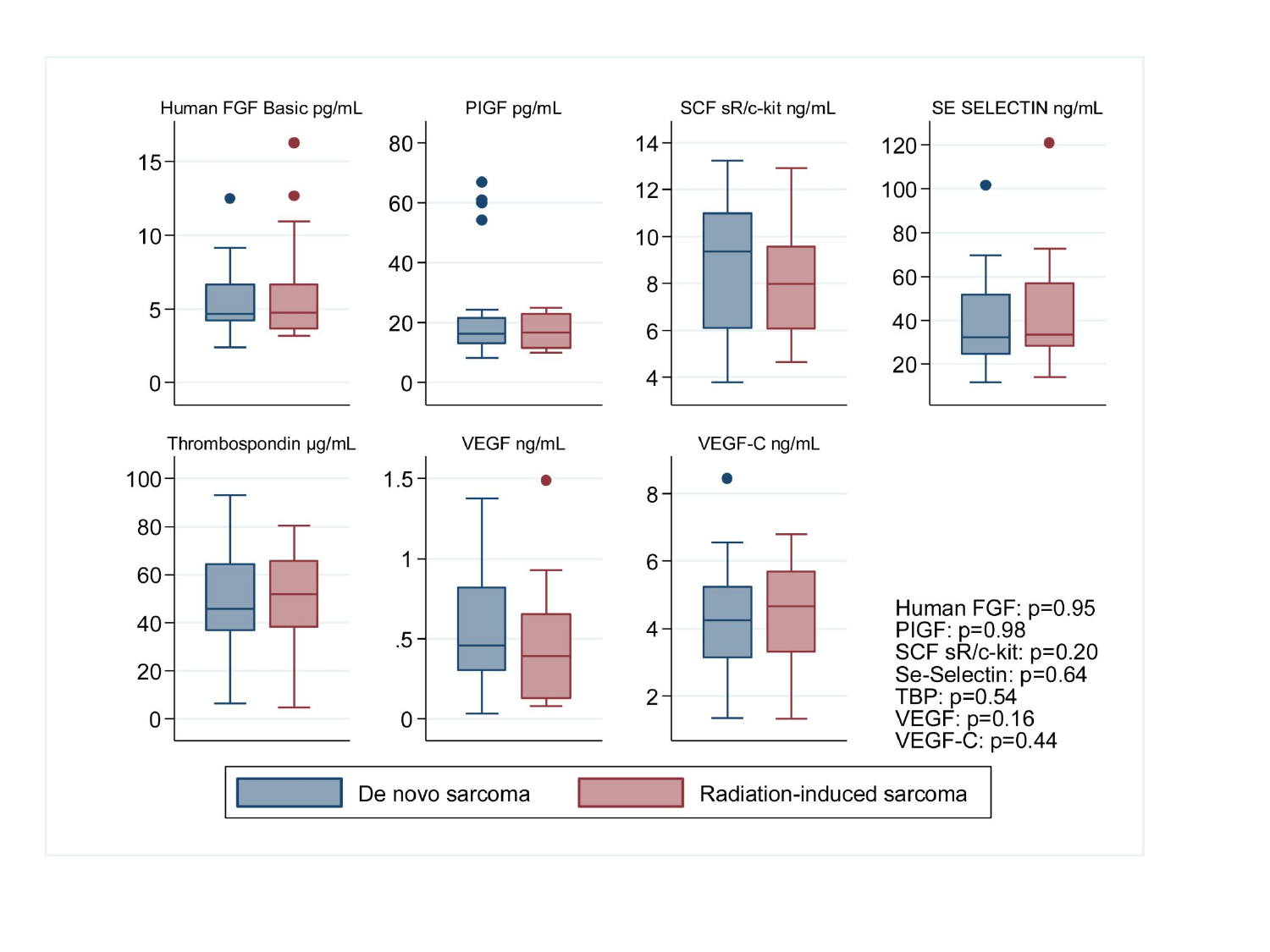

Supplement: Supplementary file 3 — Figure S2. Baseline biomarker values according to medical history (de novo versus radio-induced angiosarcoma). (PPTX 290 kb) [file 12885_2018_4828_MOESM3_ESM.pptx]

## Slide 1
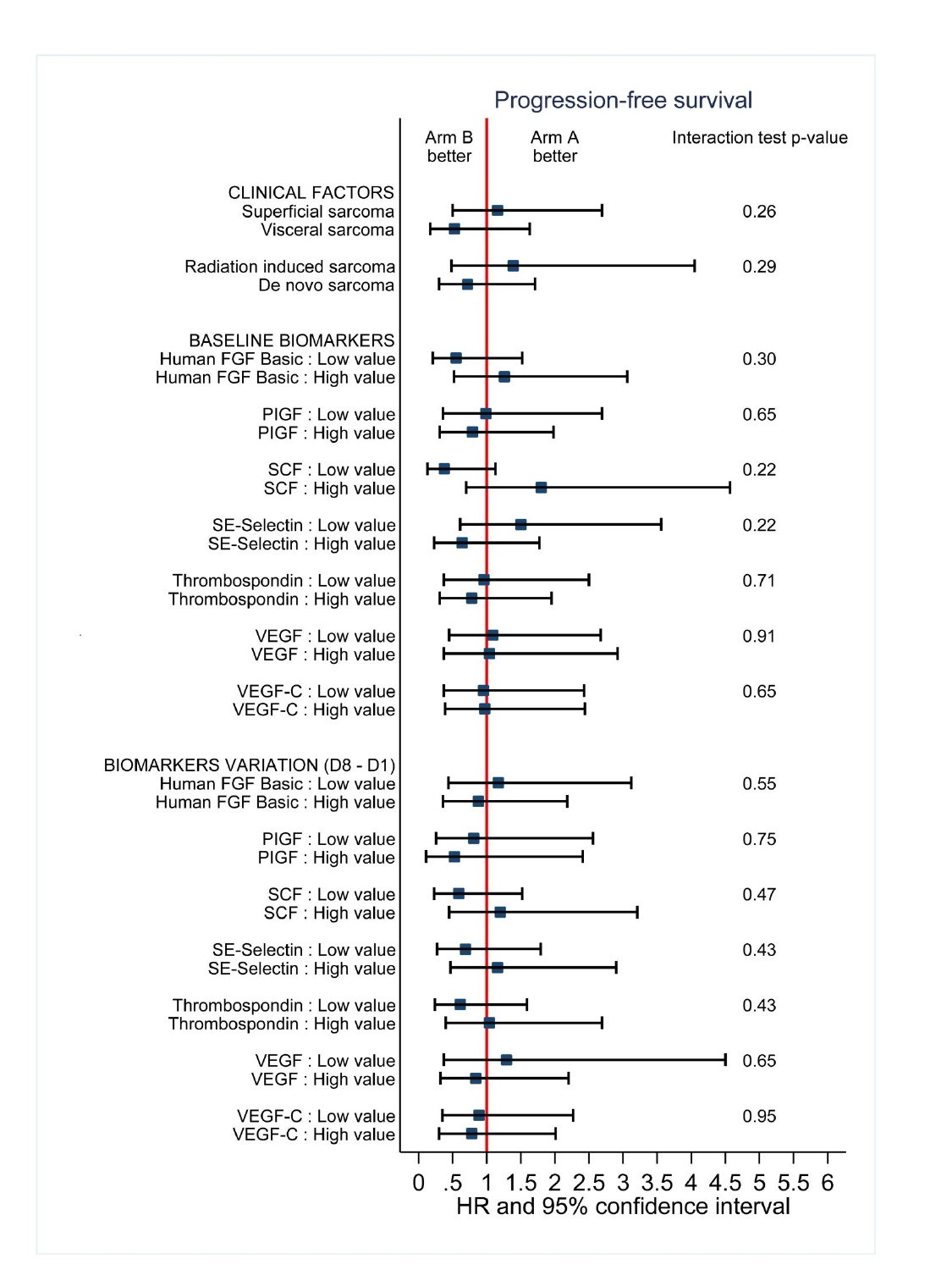

Supplement: Supplementary file 4 — Figure S3. Forest plot: evaluation of the predictive value of clinical factors and biomarkers for the treatment effect in terms of PFS. Biomarkers were categorized as binary variables using the observed median value as the cut-off to illustrate the results. (PPTX 415 kb) [file 12885_2018_4828_MOESM4_ESM.pptx]
