# Supplementary material for: Prognostic and predictive factors for angiosarcoma patients receiving paclitaxel once weekly plus or minus bevacizumab: an ancillary study derived from a randomized clinical trial
Source: BMC Cancer. 2018 Oct 11;18:963. doi: 10.1186/s12885-018-4828-1 (PMC6180490; doi:10.1186/s12885-018-4828-1)
Supplement: Supplementary file 2 — Table S1. All Grade ≥ 3 drug-related adverse events. (DOCX 13 kb) [file 12885_2018_4828_MOESM2_ESM.docx]

Supplementary Table 1 - All Grade ≥ 3 drug-related adverse events

| Toxicity | **No. (%) of Adverse Events by Grade (1)** | | | | | |
| --- | --- | --- | --- | --- | --- | --- |
|  | **Arm A (N=18)** | | | **Arm B (N=24)** | | |
|  | 3 | 4 | 5 | 3 | 4 | 5 |
| Lymphopenia | 1 (4.3) | 0 | 0 | 0 | 0 | 0 |
| Neutropenia | 2 (11.1) | 0 | 0 | 3 (12.5) | 0 | 0 |
| Fatigue | 0 | 0 | 0 | 1 (4.2) | 0 | 0 |
| Neuropathy | 0 | 0 | 0 | 1 (4.2) | 0 | 0 |
| Abdominal pain | 0 | 0 | 0 | 2 (8.3) | 0 | 0 |
| Diarrhea | 0 | 0 | 0 | 1 (4.2) | 0 | 0 |
| Intestinal occlusion | 0 | 0 | 0 | 0 | 0 | 1 (4.2) |
| Dyspnea | 0 | 0 | 0 | 2 (8.3) | 0 | 0 |
| Venous thrombosis | 0 | 0 | 0 | 0 | 1 (4.2) | 0 |
| Pulmonary embolism | 0 | 0 | 0 | 0 | 1 (4.2) | 0 |
| Cardiac failure | 0 | 0 | 0 | 1 (4.2) | 0 | 0 |
| Arterial hypertension | 0 | 0 | 0 | 1 (4.2) | 0 | 0 |
| Hemorrhagic syndrome | 0 | 0 | 0 | 2 (8.3) | 0 | 0 |
| Peritonitis | 0 | 0 | 0 | 1 (4.2) | 0 | 0 |

NOTE. Patients enrolled onto arm A received paclitaxel once per week alone; those in arm B, paclitaxel once per week plus bevacizumab. (Updated data compared to the initial paper [14])

(1) Graded according to the National Cancer Institute Common Terminology Criteria for Adverse Events, version 4.0.
